# Transfusion-associated immunomodulation: Quantitative changes in cytokines as a measure of immune responsiveness after one time blood transfusion in neurosurgery patients

**DOI:** 10.4103/0973-6247.67021

**Published:** 2010-07

**Authors:** Prashant Pandey, Rajendra Chaudhary, Amita Aggarwal, Raj Kumar, Dheeraj Khetan, Anupam Verma

**Affiliations:** *Department of Transfusion Medicine, Sanjay Gandhi Postgraduate Institute of Medical Sciences, Lucknow, India*; 1 *Department of Immunology, Sanjay Gandhi Postgraduate Institute of Medical Sciences, Lucknow, India*; 2 *Department of Neurosurgery, Sanjay Gandhi Postgraduate Institute of Medical Sciences, Lucknow, India*

**Keywords:** Cytokines production, immunologic changes, leukofiltration, soluble mediators, transfusion

## Abstract

Very few studies in humans have investigated the laboratory evidences suggestive of transfusion-associated immunologic changes. In this prospective study, we examined the effects of perioperative blood transfusion on immune response, by measuring various cytokines production, namely, interferon-gamma (IFN-γ), interleukin-10 (IL-10), and Fas Ligand (FasL). A total of 40 patients undergoing neurosurgery were randomly allocated into four groups: (a) no transfusion, (b) allogeneic non-leukofiltered transfusion, (c) prestorage leukofiltered transfusion, (d) autologous transfusion. Samples were collected before operation (day 0) and postoperative days (post-op) 1, 7, and 14. IFN-γ and IL-10 production capacity was measured in supernatant after whole blood culture and serum FasL levels in patients’ sera using commercially available ELISA kits. Change in ratios (cytokine value after PHA stimulation/control value) of IFN-γ and IL-10 and percentage change from baseline for serum FasL levels across different transfusion groups during the sampling period were calculated. There was an increase in IL-10 production in patients receiving allogeneic non-leukofiltered transfusion on days 1 and 7 (mean ratio 2.22 (± 2.16), 4.12 (± 1.71), 4.46 (± 1.97) on days 0, 1, and 7, respectively). Similarly there was a significant (*P*<0.05) decrease in IFN-γ production in patients who received allogeneic non-leukofiltered red cell transfusion on post-op days 1, 7, and 14 (mean ratio 6.88 (± 4.56), 2.53 (± 0.95), 3.04 (± 1.38) and 2.58 (± 1.48) on day 0, 1, 7, and 14, respectively). Serum FasL production was increased across all patients till 7th day except for ‘no transfusion’ group and this increase was most significant in the non-leukofiltered group. We conclude that one time transfusion leads to quantitative changes in levels of these cytokines largely through interplay of Th2/Th1 pathways in allogeneic nonleukofiltered blood transfusion; however, soluble mediators like FasL which are also present in autologous and leukofiltered blood products may contribute toward minor immunologic effect in these settings.

## Introduction

Although blood transfusion is a life-saving therapy, it is also associated with various ill effects, which can cause increased morbidity and mortality in recipients. Transfusion of blood components has been implicated to cause significant changes in recipient’s immune response, by either activating or down-regulating the immune system. The down-regulation of recipient’s cellular immune response caused by transfusion of blood (allogeneic) has been traditionally defined as transfusion-associated immunomodulation (TRIM).[1]

The clinical impact of TRIM can broadly be divided into two categories, namely beneficial (prolonged graft survival in solid organ transplants,[2,3] decreased rate of spontaneous recurrent abortions[4,5] and decreased relapse rate of inflammatory bowel disease[6]) or detrimental (increased chances of post operative infections[7,8] and cancer recurrence[7,9] and possibly a transfusion-related multiple organ dysfunction syndrome[10]).

The recognition that TRIM may be responsible for increased length of postoperative hospitalization, antibiotic usage and increased financial burden[11] in allogeneically transfused individuals has become a major concern. Despite knowing these clinical effects, the basic mechanism responsible for these observations is not known completely and disagreement among various studies has led to continued debate about its clinical relevance. TRIM is presumably mediated by allogeneic leukocytes or their soluble products,[12–14] which exert beneficial or deleterious effects in various conditions. One of the plausible mechanisms put forward is immune deviation toward T-helper lymphocytes type 2 (Th2) cytokine characterized by secretion of IL-4, IL-5, IL-10 cytokines with reduced secretion of T-helper lymphocytes type 1 (Th1) cytokines namely IL-2, IL-12, and IFN-.[5,15–17]

A majority of the studies conducted on this subject have evaluated only the deleterious clinical consequences of transfusion[18–21] and very few studies in humans[17,22] have investigated the laboratory evidences suggestive of TRIM.

In this prospective study, we examined the effect of peri-operative blood transfusion on cellular immune response by measuring changes especially in cytokine production capacity of T helper cells (IFN-γ, IL-10) and changes in serum Fas Ligand (FasL) levels in patients undergoing neurosurgical procedures.

## Materials and Methods

This randomized control study was carried out after obtaining approval from institute ethics committee at the Department of Transfusion Medicine, Sanjay Gandhi Postgraduate Institute of Medical Sciences, Lucknow, India, over a period of 2 years from January 2005 to January 2007.

### Patient Selection

A total of 40 neurosurgical patients who were likely to receive only one time transfusion of packed red blood cells in their peri-operative period with a likely follow up of 1 month were included in the study after obtaining informed consent. Patients on immunosuppressive therapy were excluded from the study. Transfusion in these 40 patients was done after randomly allocating them into four different groups (10 in each group). These four groups were (a) no transfusion, (b) allogeneic red cell transfusion, (c) pre-storage leukofiltered allogeneic red cell transfusion, and (d) autologous red cell transfusion.

The patients were randomized before surgery into four groups and they remained in that very group till the end of study period. One patient from the “no transfusion” group had to be transfused peroperatively and thus was excluded from the study; however, the total number of patients did not change as one new patient was allocated to the “no transfusion” group.

### Data Collection

The relevant clinical history of the patients namely age, gender, diagnosis, and operative details e.g. duration of surgery and type of anesthesia were obtained from the hospital information system (HIS). Details of patients’ transfusion history including the type of red cell transfusion (RBC), total number of units transfused, etc. were collected from the blood bank files.

### Red Cell Preparation

Allogeneic red cell components (buffy coat depleted) were prepared from whole blood collected in quadruple blood bags (Terumo Penpol, Trivandrum, India) using semi-automated component extractor (T-ACE, Terumo, Teruflex). Prestorage leukocyte depletion was performed on packed red blood cells using red blood cell filters (Imugard III-RC, Terumo, Japan). In autologous group all patients donated 2 units preoperatively (350 mL whole blood per donation) with a gap of 7 days in between and last unit collected at least 3 days before the operation. The autologous whole blood units were also processed into red cell components following the departmental standard operating procedure.

### Sample Collection

The sampling protocol was common to all the four groups included in the study. From each patient 2 mL sample in a heparinized vial for the estimation of IL-10 and IFN-γ and 2 mL sample in a plain vial for serum FasL estimation were collected before the beginning of operation (day 0) and post operative days (post-op) 1, 7, and 14.

### Cytokine Estimation

#### Whole blood culture for supernatant generation (IL-10 and IFN-γ)

IL-10 and IFN-γ were measured in the supernatant obtained after the whole blood cell culture technique for measurement of stimulated cytokine secretion.[23] Briefly, heparinized whole blood was diluted 1:5 with complete Roswell Park Memorial Institute (cRPMI-1640) media containing antibiotic and antimycotic (Sigma Chemicals, Germany). For stimulation, 5 μg/mL concentration of phytohemagglutinin (PHA) was used. The supernatant of unstimulated cell culture (without PHA) was considered as negative control. Both stimulated and unstimulated cell culture tubes were loosely covered and incubated in a humidified atmosphere of 5% CO_2_ at 37°C for 48 h. After completion of incubation, culture supernatant was harvested carefully and stored frozen at –70°C.

#### Measurement of IL-10, IFN-γ

Production of IL-10 and IFN-γ in stored whole blood culture supernatants was measured by commercially available ELISA kits following the manufacturer’s instructions (IL-10 ELISA kits from BD Phar Mingen San Diego, CA; sensitivity 7.8 pg/mL) and IFN-γ (R&D, USA; sensitivity 15.6 pg/mL).

#### Measurement of Serum FasL

The FasL levels were measured on patients’ sera till day 7 only by commercially available Duoset ELISA kit (R&D, USA, sensitivity 31.25 pg/mL) following the manufacturer’s instructions. The 14 day samples were not available for the FasL assay.

### Statistics

Statistical analysis was done using a statistical software package (SPSS version 12). The mean values (±S.D.) for cytokine production in case of IFN-γ and IL-10 were calculated. As baseline mean values for cytokines differed among various groups, change in ratios (cytokine value after PHA stimulation / unstimulated control value) of IFN-γ and IL-10 and percentage change from baseline for serum FasL levels across different transfusion groups during the sampling period were estimated. The percentage change from baseline for Fas L was evaluated using the formula

$$\%\;\mathrm{Change}\;\mathrm{in}\;\mathrm{cytokine}\;\mathrm{level}\;=\;\frac{(\mathrm{Level}\;\mathrm{on}\;\mathrm{sampling}\;\mathrm{day}\;–\;\mathrm{Level}\;\mathrm{on}\;\mathrm{day}\;0)\;\;\times \;100}{\mathrm{Level}\;\mathrm{on}\;\mathrm{day}\;0}$$

The difference in cytokine production on different days within groups was determined using repeated measures of the non-parametric test (Friedman test). *P* value <0.05 was considered as significant.

## Results

The clinical details of patients selected for the study are shown in Table 1. Prolapsed intervertebral Disc (PIVD) was the main cause for undergoing surgery in 19 out of 40 patients. All the patients received packed red blood cell transfusion only once during the operation and none received blood transfusion in the post operative period. A total of 29 red cell units (mean 2.9 units) were transfused to those patients who received allogeneic non-leukofiltered units. While 15 units (mean 1.5 units) were transfused to those who received leukofiltered red blood cells. Patients of the autologous blood transfusion group received a total of 19 units (mean 1.9 units).

**Table 1:** Clinical characteristics of neurosurgery patients under study (n=40)

Characteristics Median age in years (range) Gender (M / F)	Number 35 (8-62) 34 / 6			

Diagnosis	No transfusion	Autologous	Allogeneic non-LF	Allogeneic LF
Arnold Chiari malformation (01)	--	--	--	01
PIVD and/or myelopathy (19)	07	05	03	04
Traumatic nerve injury	01	01	01	--
CP angle epidermoid cyst	--	--	--	01
Pott’s spine	01	--	01	01
Paraganglioma	--	--	--	01
Frontal abscess with osteomyelitis	--	--	01	01
Lumbar canal stenosis	--	01	01	01
Glioma	--	01	01	--
Ependymoma	--	01	01	--
Subarachnoid hemorrhage	01	--	01	--
Spastic paraparesis	--	01	--	--

### 

#### Cytokine Production Capacity in Different Study Groups

### IFN-γ [Table 2]

**Figure 1:**
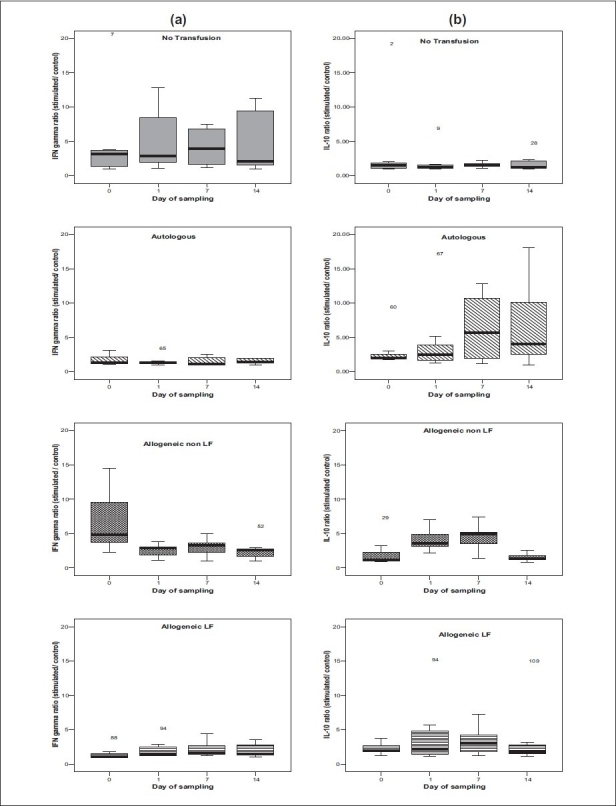
Change in ratios (cytokine value after PHA stimulation / control values) of IFN-γ (a) and IL-10 (b) across different transfusion groups during the sampling period

There was no significant change over the study period in IFN-γ production in patients who received no transfusion or receiving allogeneic leukofiltered or autologous red cell transfusion. However, there was a significant (*P*<0.05) decrease in IFN-γ production in patients who received allogeneic non-leukofiltered red cell transfusion on post-op days 1, 7, and 14 compared to baseline production [mean ratio 6.88 (± 4.56), 2.53 (± 0.95), 3.04 (± 1.38) and 2.58 (± 1.48) on day 0, 1, 7, and 14, respectively] [Figure 1a].

**Table 2:** IFN-γ and IL-10 production across different groups during the sampling period

Group	Day 0			Day 1			Day 7			Day 14		
	U*	S**	R#	U	S	R	U	S	R	U	S	R
	
	IFN-γ											
No transfusion	60.98 (70.5)	150.75 (125.68)	4.92 (6.79)	72.59 (68.19)	251.96 (184.95)	6.91 (8.38)	93.26 (96.21)	242.17 (202.41)	4.22 (2.82)	86.78 (105.89)	217.14 (151.45)	5.17 (4.70)
Autologous	129.79 (101.10)	188.26 (96.99)	1.78 (0.75)	120.88 (90.28)	171.22 (130.41)	1.47 (0.63)	141.47 (104.79)	185.42 (96.65)	1.57 (0.67)	149.56 (96.85)	203.09 (103.79)	1.55 (0.39)
Allogeneic non-LF##	144.82 (112.17)	636.95 (147.08)	6.88 (4.56)	145.67 (90.95)	307.56 (93.83)	2.53 (0.95)	127.92 (87.67)	316.97 (128.51)	3.04 (1.38)	156.79 (77.06)	339.40 (140.44)	2.58 (1.48)
Allogeneic LF IL-10	32.62 (13.51)	44.51 (18.21)	1.5 (0.83)	27.88 (10.60)	55.84 (28.05)	2.13 (1.25)	28.07 (11.08)	62.54 (38.91)	2.21 (1.16)	29.04 (13.68)	67.79 (63.96)	2.10 (1.01)
No transfusion	159.66 (168.25)	213.55 (156.56)	3.84 (6.54)	173.65 (159.64)	233.35 (173.74)	1.98 (1.95)	84.10 (73.51)	113.05 (71.15)	1.62 (0.39)	120.61 (109.12)	194.06 (139.95)	1.83 (1.12)
Autologous	48.34 (32.41)	117.81 (62.18)	3.06 (2.59)	53.52 (49.91)	126.45 (68.56)	4.51 (5.50)	32.68 (31.67)	134.62 (81.50)	6.43 (4.93)	28.47 (33.26)	110.47 (94.90)	6.91 (6.20)
Allogeneic non-LF##	50.33 (33.45)	68.99 (25.22)	2.22 (2.16)	79.03 (43.60)	280.90 (88.29)	4.12 (1.71)	87.19 (76.20)	278.57 (53.82)	4.46 (1.97)	46.67 (16.86)	70.16 (35.26)	1.52 (0.61)
Allogeneic LF	31.92 (19.31)	71.67 (52.60)	2.29 (0.90)	31.51 (24.57)	74.84 (33.01)	4.34 (4.81)	17.27 (17.97)	41.95 (22.37)	3.37 (2.09)	38.31 (38.76)	74.94 (44.92)	3.73 (4.79)

* U- Un-stimulated cytokine production (pg/mL);

** S - Cytokine production after PHA stimulation (pg/mL);

# R - Ratio of stimulated to un-stimulated cytokine production;

## - Significant difference in cytokine production over the sampling period *P*<0.05, Friedman test)

### IL-10 [Table 2]

Similar to *IFN-γ*, there was no significant change over the sampling period in IL-10 production in patients who received no transfusion or received allogeneic leukofiltered or autologous red cell transfusion. However, there was a significant (*P*<0.05) increase in IL-10 production in patients who received allogeneic non-leukofiltered red cell transfusion on days 1 and 7 compared to baseline [mean ratio 2.22 (± 2.16), 4.12 (± 1.71), 4.46 (± 1.97) on days 0, 1, and 7, respectively]. The values returned to normal on post-op day 14 [Figure 1b].

### FasL

**Figure 2:**
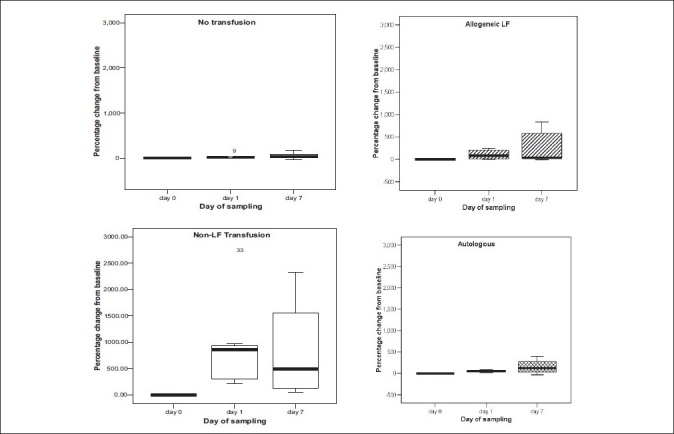
Percentage change in serum FasL production in different study groups till day 7 post-op

Unlike other cytokines, there was a significant (*P*<0.05) change in serum FasL levels in patients across all the groups till 7th post-op day compared to baseline except for ‘no transfusion group’ where the serum FasL levels normalized on post-op day 7. An increase in FasL levels was most significant in the non-leukofiltered group where median percentage change from baseline was 860% (range 208 to 2666) and 493.13% (range 48 to 2332) on post-op days 1 and 7, respectively [Figure 2].

## Discussion

The cellular immune response is of paramount importance in surgical settings particularly in the perioperative period. The cytokine dysregulation has been proposed as the potential mechanism for transfusion-associated immunomodulation.[16,17,24]

In the present study, we examined the influence of allogeneic red cell, leukofiltered red cell, and autologous red cell transfusions on cytokine production as a functional measure of immunologic changes in otherwise healthy neurosurgery patients. Patients undergoing neurosurgical procedures (majority having PIVD) were included in this study as most of them received single time peri-operative red blood cell transfusions only, unlike patients with malignancy of gastrointestinal tract[8,9,19,25] or cardiac surgery patients[20,21,26] who are the most commonly studied patient groups in the literature for host immune responsiveness.

We studied the effect of leukodepletion on the production of cytokines of Th1 (IFN-γ) and Th2 (IL-10) pathways till 14 days postoperatively. There was no significant change over the sampling period in IL-10 and IFN-γ production in patients who received no transfusion or received allogeneic leukofiltered or autologous red cell transfusion. However, there was a significant increase in mean values from baseline in IL-10 production in patients who received allogeneic non-leukofiltered red cell transfusion on days 1 and 7. The values returned to normal on post-op day 14 [Figure 1b]. Similarly there was a significant (*P*<0.05) decrease in IFN-γ production in patients who received allogeneic non-leukofiltered red cell transfusion on post-op days 1, 7, and 14 compared to baseline [Figure 1a].

Similar results were found in a studywhere authors studied IL-4 and IL-10 of the Th2 pathway and IL-2 of the Th1 pathway and observed the changes in these cytokine levels from baseline to multiple postoperative samples after major joint replacement surgery.[17] In 14 patients, who received allogeneic non-leukofiltered transfusion, a remarkable increase in both IL-4 (5 times of pre-op levels) and IL-10 (15 times of pre-op levels) was seen as compared to those patients who received either no transfusion or autologous transfusion. No significant change in the mean levels of TH1 cytokine (IL-2) was seen in any of their patient groups. In an another study conducted on a mouse model to demonstrate the effect of blood transfusion on cytokine production by Th1 and Th2 pathways following a single allogeneic blood transfusion, a difference in lymphokine production was observed compared to controls.[15] Cytokines of the Th2 pathway (IL-4, IL-10) were produced at higher level, while production of cytokine of the Th1 pathway (IL-2) was significantly reduced. They found a steady increase in IL-4 production in animals transfused with blood at 14 days after transfusion. IL-10 production peaked sharply at 3 days after transfusion (5 vs 2.6 unit/mL for transfused and control animals, respectively) and then declined to control levels by 7 days after transfusion. In contrast to IL-4 and IL-10, the production of IL-2 from transfused animals was significantly lower than production from controls. Production of IL-2 remained significantly suppressed in transfused animals throughout the study period (14 days after transfusion). A study done in 1992 also demonstrated increased IL-4 and IL-10 transcripts in the graft and spleen using a murine heart allograft model.[27] Similarly in randomized controlled trials comparing the effects of allogeneic blood transfusion versus autologous blood transfusion, a statistically significant increase was observed in mean levels of cytokines of the Th2 pathway (IL-10) in allogeneic transfusion arm which remain increased up to day 7 post-op.[9]

An important observation in our study was the occurrence of baseline variation in cytokine production among the different groups which is similar to the findings of Bordin and coworkers[28] who, in a study involving 47 patients (allogeneic nonfiltered RBC group= 17, filtered RBC group= 14, and untransfused group=16) undergoing hip replacement surgery, noted preoperatively biologic differences among patients in the baseline level of cytokines produced in response to PHA stimulation. They attributed the variation in cytokine secretion to the variable numbers of leukocytes (WBCs) present in the blood cultures, reflecting the patients’ leukocyte counts. However, as regards to the changes in cytokine production our results are in partial agreement with their findings as about 30% of their patients transfused with allogeneic RBCs (non-leukofiltered) had a decrease in IL-2 production and about 8% of the patients transfused with WBC-reduced RBCs (pre-storage leukofiltered) had a decrease in IL-2 production on days 1 and 4 after surgery. In addition, they found that about 70% and 35% of the patients transfused with allogeneic RBCs showed a decrease of at least 20% in the production of IFN-γ and TNFα, respectively on day 4. Thus, production of these cytokines of the Th1 pathway was decreased in non-leukofiltered allogeneic blood transfusion although unlike our findings no significant differences were detected in the *in vitro* production of Th2-type cytokines (lL-6 and IL-10). In our study the deviation in cytokine production of the Th1 and Th2 pathways was associated with transfusion of allogeneic non-leukofiltered blood and was conspicuously absent in leukofiltered, autologous, and no transfusion groups; thus, it can be surmised that contaminating allogeneic leukocytes present in non-leukofiltered blood could be responsible for this effect.

We observed that the whole blood assay was simple, effective, and less time consuming for production of cytokines instead of separation of peripheral blood mononuclear cells. This assay offered the opportunity to asses a white cell distribution that is normally present *in vivo*, and it allowed interactions between blood components that must also be considered for the *in vivo* conditions.

The Fas/(FasL) system has been recognized as a major pathway for the induction of apoptosis in cells and tissues. The T-cell apoptosis induced by Fas/FasL binding serves to down-regulate the immune response to antigens. The serum FasL levels are reported to be increased in patients with various clinical conditions, namely sepsis,[29] viral infections,[30,31] and disseminated intravascular coagulation.[32] Also concentrations of FasL and other soluble molecules in the supernatant of blood units have been found to be increased in stored blood units. It is suggested that these soluble molecules are derived from leukocytes present in the unit which may exert immunomodulation.[33] Experimental evidence exists that soluble mediators, for example, HLA molecules, FasL, can cause TRIM in an autologous *in vitro* setting.[14] But to the best of our knowledge there are no studies available in the literature for comparing serum FasL in patients on various transfusion protocols. Serum FasL production was increased across all patients till 7th day except for the ‘no transfusion’ group and this increase was most significant in the non-leukofiltered group [Figure 2]. The results show that increased concentrations of soluble mediators like FasL are found in the recipients even after prestorage leukofiltered allogeneic or autologous blood transfusions. The cytokine assay in the recipients before and after red cell transfusion along with cytokine level in the donor unit could have accurately reflected *in vivo* changes in these patients. Nonetheless, our study demonstrates that allogeneic and autologous transfusion may exert similar immunologic changes due to involvement of soluble mediators derived probably from leukocytes.

Surgery also contributes to the deviation of the immune response toward a Th2 and patients undergoing surgery and anesthesia have shown to present with down-regulated IL-2 secretion and up-regulated IL-10 and IL-4 secretion during the postoperative period.[34] However, in our study these confounding factors did not play much role as all patients underwent surgical procedures of almost similar duration, same type of anesthesia and single team of surgeons.

To evaluate the clinical surrogate markers of immunologic changes after transfusion, we collected the patients’ details (results not shown) as regard to postoperative infections and length of hospital stay (LOS) and we found that 23% of patients who received non-leukofiltered allogeneic blood transfusion experienced postoperative bacterial infections (local wound infection), while those patients who received leukofiltered blood transfusion, autologous blood transfusion, and no blood transfusion experienced no such deleterious effects. The mean LOS in allogeneic non-leukofiltered was 9.14 days compared to 7.55, 7.43, 7.3 in leukofiltered, autologous, and no transfusion groups, respectively. This is explained by the finding that post-op infection was observed only in allogeneic non-leukofiltered group, which may be responsible for increased LOS. These observations are in agreement with other published works.[17,35,36]

The study has certain limitations such as small sample size, unavailability of cytokine production in donor units, and lack of data for functional or qualitative immunological alterations. Furthermore, as the baseline values for cytokines differed greatly across different study groups, we could not evaluate between-group differences (inter-group comparison) in cytokine production. It is inherent in such studies that not all biases can be removed completely which directly or indirectly affect the laboratory parameters and clinical outcomes. It is expected that multicentric double blind randomized control trials will reveal strong unequivocal evidences for transfusion immunomodulation.

In conclusion, the results of this study show that one-time transfusion during the perioperative period in neurosurgical patients leads to quantitative alteration in specific cytokine production mainly through interplay of the Th2/Th1 pathways of immune response indicating role of leukocytes or their soluble products as the major mediators of immunologic changes. Additionally, the possibility of altered immune response due to soluble mediators such as FasL after transfusion of allogeneic leukofiltered blood and/or autologous blood still exists. These changes in cytokine patterns may contribute to increased morbidity in patients even after one-time blood transfusion.
